# A case series of medically managed *Candida parapsilosis* complex prosthetic valve endocarditis

**DOI:** 10.1186/s12941-020-00409-4

**Published:** 2021-01-05

**Authors:** Penghao Guo, Yuting He, Rui Fan, Zhongwen Wu, Yili Chen, Yuli Huang, Kang Liao, Peisong Chen

**Affiliations:** 1 Department of Clinical Laboratory, The First Affiliated Hospital, Sun Yat-Sen University, 58 Zhongshan road II, Guangzhou, Guangdong China; 2 Department of Ultrasound, Institute of Diagnostic and Interventional Ultrasound, The First Affiliated Hospital, Sun Yat-Sen University, Guangzhou, China; 3 Clinical Medicine Research Center, Shunde Hospital, Southern Medical University, Foshan, People’s Republic of China

**Keywords:** Prosthetic valve endocarditis, *Candida parapsilosis* complex, Combination antifungal therapy, Matrix-assisted laser desorption ionization-time of flight mass spectrometry

## Abstract

**Background:**

In recent years, *Candida parapsilosis* is recognized as a species complex and is composed of *Candida parapsilosis *sensu stricto, *Candida orthopsilosis* and *Candida metapsilosis*. *Candida parapsilosis* complex prosthetic valve endocarditis (PVE) is rare and the survival rate is still low despite of optimal therapeutic strategies. In our report, it is novel to report cases as *Candida parapsilosis* complex PVE at species and identify *Candida parapsilosis* using MALDI-TOF MS.

Case presentation

A series of 4 cases of *Candida parapsilosis* complex PVE from our institution was reported. Three were infected by *Candida parapsilosis *sensu stricto and one was infected by *Candida metapsilosis.* The condition of two cases got better and the other died.

**Conclusions:**

More attention should be paid to *Candida parapsilosis* complex PVE and early diagnosis and prompt antibiotic therapy may play a role in the treatment for *Candida parapsilosis* complex PVE. It is recommended to identify *Candida parapsilosis* complex at species level and MALDI-TOF MS as an easy, fast and efficient identification method is worth promoting in clinical microbiology

## Introduction

Prosthetic valve endocarditis(PVE) is a complication of cardiac valve replacement and is related with a high mortality [1]. What’s more, the incidence of PVE is increasing and it accounts for 20–30% of infective endocarditis episodes [2]. Generally, typical microorganisms causing PVE were mainly bacteria, especially *Enterococci* and *Staphylococcus aureus* [3]. Fungal endocarditis(FE) is a rare and fatal form of infectious endocarditis [4]. *Candida* and *Aspergillus* species are two of the most common etiologic fungi for FE. Among *Candida* endocarditis, *Candida albicans* is the main cause of FE and *Candida parapsilosis* is the most common non-albicans species responsible for FE [5]. In the last years, *Candida parapsilosis* is recognized as a species complex and is composed of *Candida parapsilosis *sensu stricto, *Candida orthopsilosis* and *Candida metapsilosis*, which are unique but related [6]. The *Candida parapsilosis* complex is opportunistic fungal pathogen responsible for many human nosocomial infections. *Candida parapsilosis* complex PVE is rare and the survival rate is still low despite of optimal therapeutic strategies [7]. However, the literature on *Candida parapsilosis* complex PVE is limited. What’s more, the previous studies did not identify *Candida parapsilosis* complex at species level. More information about *Candida parapsilosis* complex PVE at species level and the management of it is needed. Here we present a case series of *Candida parapsilosis* complex PVE in patients with aortic valve replacement (AVR) or mitral valve replacement (MVR) from the First Affiliated Hospital of Sun Yat-Sen University, providing more information about *Candida parapsilosis* complex PVE at species level and reference on the treatment of it. All patients received detailed counseling and informed written consent was obtained from each participant.

## Case description

### Case report 1

**Fig. 1:**
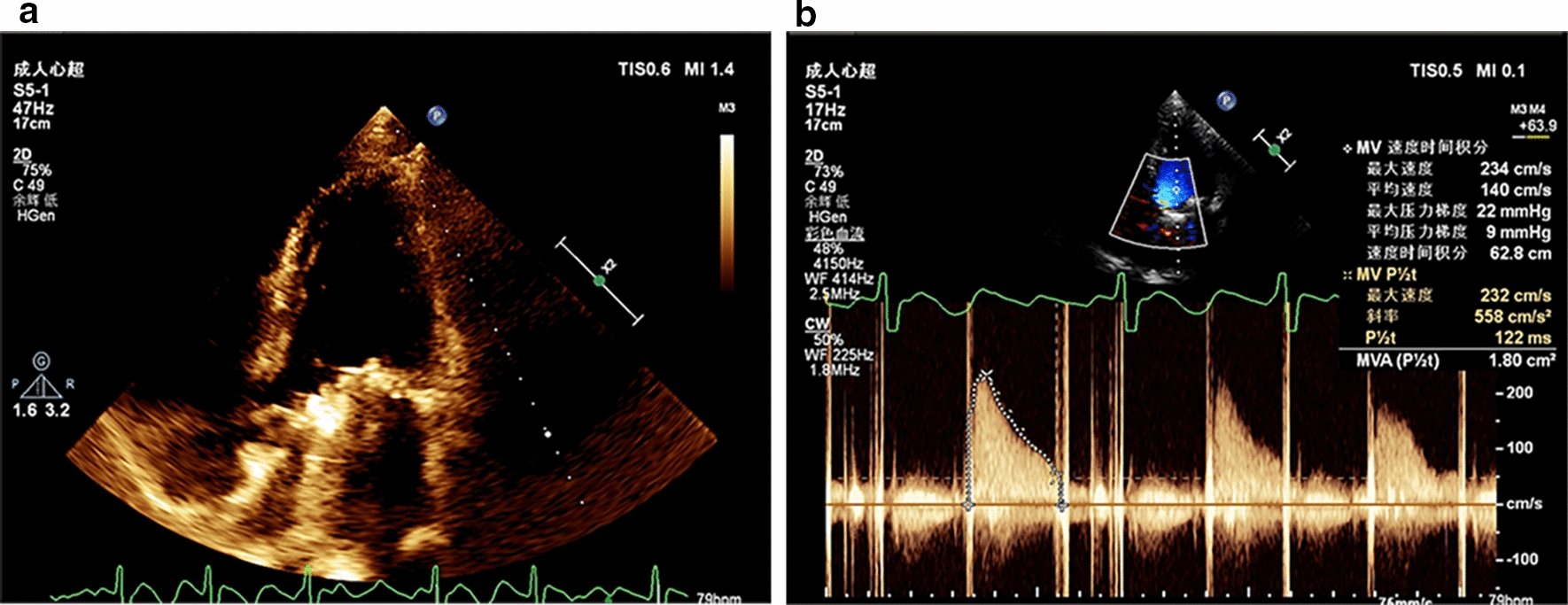
The *Candida parapsilosis* complex prosthetic valve endocarditis of case 1. **a** Echocardiography showed a vegetation on the artificial mitral valve. **b** The Color Doppler showed increased flow rate at the prosthetic mitral valve orifice and tricuspid regurgitation (moderate)

The first patient was a 55-year-old man who had a history of smoking for more than 10 years. The patient underwent mitral valve replacement in 2012. On 24th of December 2017, he was admitted into the First Affiliated Hospital of Sun Yat-Sen University because of aggravated shortness of breath for one month and edema of both lower extremities for 3 days. The initial blood count showed hemoglobin level of 100 g/L, white blood cell (WBC) count of 5.28 × 10^9^ cells/L (46.6% neutrophils, 36.2% lymphocytes), raised erythrocyte sedimentation rate (ESR) (74 mm/h) and C-reactive protein (CRP)(10.60 mg/L). The procalcitonin (PCT), troponin T and N-terminal prohormone of brain natriuretic peptide (NT-proBNP) all increased (Table S1). Bed-side chest radiograph showed inflammation of both lungs and enlarged heart shadow in the supine position. Transthoracic echocardiography (TTE) showed two vegetations on prosthetic mitral valve and accelerated velocity of the prosthetic mitral valve (140 cm/s) The effective orifice area was 1.8 cm^2^ (Fig. 1), indicating infectious endocarditis after MVR. TTE also found moderate tricuspid regurgitation and mild pulmonary artery hypertension. The left ventricular ejection fraction (LVEF) was approximately normal (56%) and the diastolic function was reduced. The electrocardiograph (ECG) indicated atrial flutter (2–3: 1 conduction) with rapid ventricular rate. The (1,3)-β-D glucan was 93.99 pg/mL and *Candida parapsilosis *sensu stricto was identified in the blood culture by Matrix-assisted laser desorption ionization-time of flight mass spectrometry (MALDI TOF–MS) (bioMerieux, France) (Flucytosine minimal inhibitory concentration (MIC) 4 ug/mL, amphotericin B MIC 0.5 ug/mL, voriconazole MIC 0.06 µg/mL, itraconazole MIC 0.125 µg/mL, fluconazole MIC 1 µg/mL). The cutoff values for antifungal susceptibility testing were on the basis of CLSI M59 and CLSI M60 [8, 9]. Repeat blood cultures continued to grow *Candida parapsilosis *sensu stricto. The patient was initiated on vancomycin 1 g iv three times a day and voriconazole 200 mg iv twice a day after admission. Then, combination antifungal therapy with vancomycin 1 g iv three times a day and caspofungin iv 50 mg once a day was initiated on Dec 29. Because of high vancomycin blood concentration, the therapy changed to vancomycin 1 g iv twice a day and caspofungin 50 mg iv once a day on Dec 30 and lasted until discharging. Besides, ceftriaxone sodium was administrated from Dec 30 of 2017 to Jan 3 of 2018 to fight lung infections. After a series of anti-infective treatment, the patient's condition improved. However, the blood cultures continued to grow *Candida parapsilosis *sensu stricto and the patient refused the surgical treatment despite of the indications of operation. The patient went back to local hospital and was recommended to continue the combination antifungal therapy according to the drug sensitivity.

### Case report 2

The second patient was a 71-year-old man with a history of hypertension for 3 years and smoking for 30 years. He was admitted into the First Affiliated Hospital of Sun Yat-Sen University on April 26 of 2019 for repeated chest tightness and palpitations for more than 10 years, aggravating by 1 year. TTE showed that posterior mitral valve tendon cord was ruptured, which resulted in posterior mitral valve prolapse and severe mitral valve regurgitation. At the same time, TEE found mild aortic valve stenosis and mild-moderate regurgitation, anterior tricuspid valve prolapse and medium regurgitation, severe pulmonary artery hypertension. The aorta root, the left atrium and left ventricle were significantly enlarged. The right atrium was slightly larger and the left ventricular ejection fraction (LVEF) was about 70%. On May 5, the patient underwent aortic valve and mitral valve bioprosthesis replacement, tricuspid valvuloplasty, aortic annuloplasty and temporary cardiac pacemaker implantation. The patient was given cefperazone-sulbactam 3 g iv three times a day from May 5 to May 17, cilastatin sodium/imipenem iv 1 g once a day from May 20 to May 28 and cefperazone-sulbactam 3 g iv three times a day from May 28 to May 31. On May 24, the blood culture grew yeast-like fungus and the yeast-like fungus was identified *Candida parapsilosis *sensu stricto by MALDI TOF–MS (bioMerieux, France) (Flucytosine MIC 4 ug/mL, amphotericin B MIC 0.5 ug/mL, voriconazole MIC 0.06 ug/mL, itraconazole MIC 0.125 ug/mL, fluconazole MIC 1 µg/mL). Then the patient was administrated caspofungin (50 mg iv once day) to fight fungal infection. On June 13, the blood culture still grew *Candida parapsilosis *sensu stricto and the patient continued to use caspofungin (50 mg iv once day). On June 24, the patient had a fever of 39.2℃ and the blood culture still *Candida parapsilosis *sensu stricto. Then the patient was given voriconazole (200 mg oral twice a day) instead of caspofungin. The blood culture became negative after 1 month using voriconazole. The patient continued to use voriconazole (200 mg oral once a day) for 2 weeks after discharging.

**Fig. 2:**
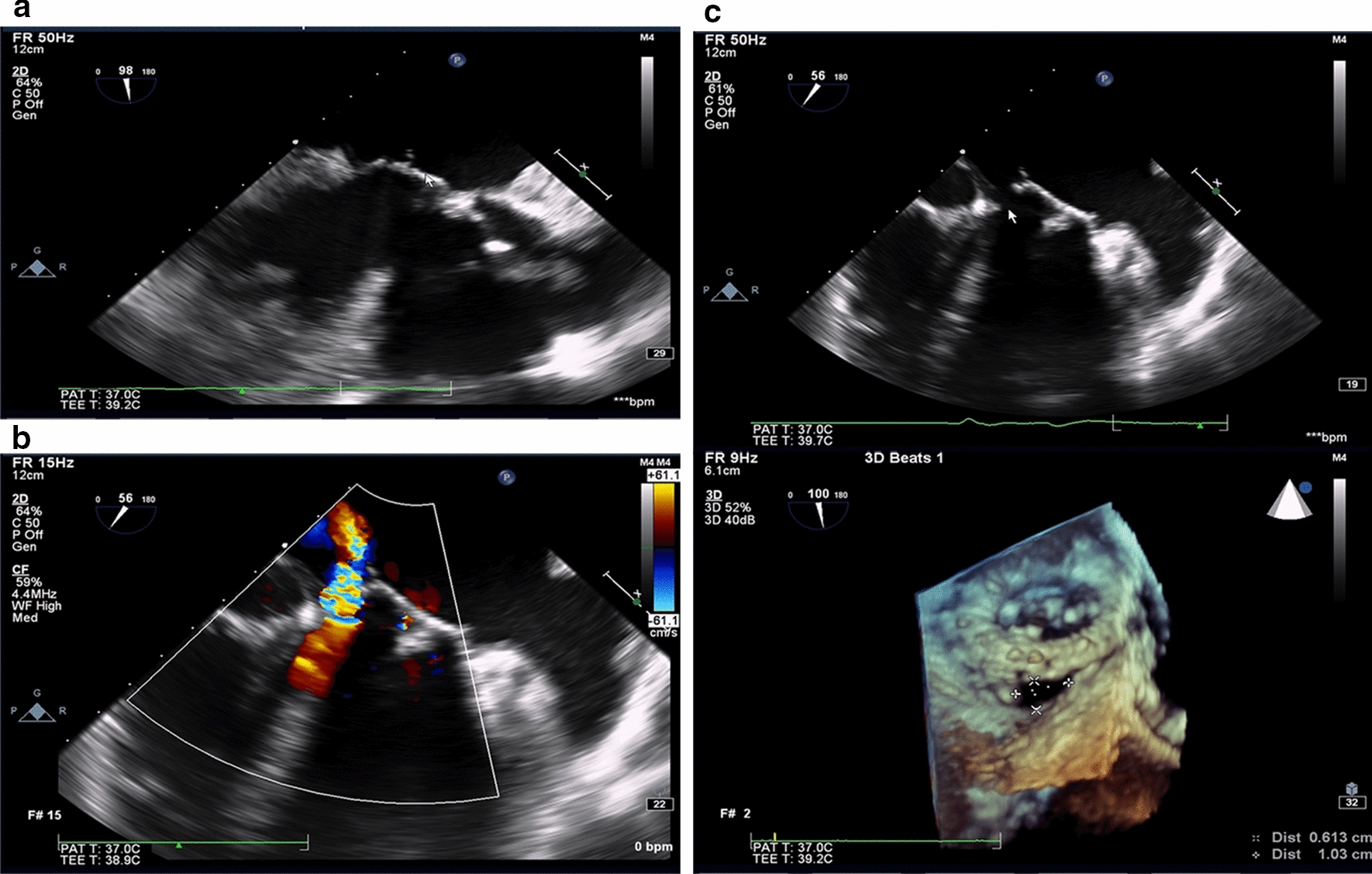
The *Candida parapsilosis* complex prosthetic valve endocarditis of case 2. **a** Echocardiography showed a short abnormal strip about 5mm×3mm on the prosthetic mitral valve of the left atrium side; **b** The Color Doppler showed perivalvular leakage (moderate-severe); **c** Transesophageal 3D image showing a bioprosthetic mitral valve. A perivalvular crack about 10.3×6.1 mm could be seen around the medial side of the prosthetic mitral valve

Six months later, the patient was readmitted into the First Affiliated Hospital of Sun Yat-Sen University on November 4 due to fever for 20 days and shortness of breath for 3 days after activity. The initial blood count showed WBC count of 3.76 × 10^9^ cells/L (78.8% neutrophils, 10.3% lymphocytes). The (1,3) -β-D glucan was 144.85 pg/mL and the blood culture grew *Candida parapsilosis *sensu stricto which was identified by MALDI TOF–MS (bioMerieux, France) (Flucytosine MIC 4 µg/mL, amphotericin B MIC 0.5 ug/mL, voriconazole MIC 0.06 µg/mL, itraconazole MIC 0.125 ug/mL, fluconazole MIC 1 ug/mL). (Table 1 and Additional file 1: Table S1). TEE showed a dehiscence about 10.3  × 6.1 mm and severe perivalvular leakage from the medial part of the prosthetic mitral valve. TEE also found a small strip fluttering a lot from left atrium side of the medial prosthetic ring, suggesting infective endocarditis (Fig. 2). The patient was administrated voriconazole 200 mg iv twice a day from November 4 to 21, caspofungin 50 mg iv twice a day from November 21 to December 4 and amphotericin B 1 mg iv once a day from November 26 to December 4. Besides, the patient was administrated piperacillin-tazobactam 4.5 g iv three times a day from Nov 4 to Nov 6, vancomycin 0.5 g iv once a day from Nov 17 to Nov 21 and cilastatin sodium/imipenem iv 1 g once a day from Nov 21 to Dec 4. The patient's body temperature was relieved, fluctuating around 37.5 ℃. Then the patient was discharged and recommended antifungal treatment in local hospital with cilastatin sodium/imipenem for injection 1000 mg three times a day, caspofungin 50 mg once a day and amphotericin B 30 mg once a day.

**Table 1:** Clinical characteristics of the patients

	Case 1	Case 2	Case 3	Case 4
Age(years)	55	71	70	64
Gender	Male	Male	Male	Male
Type of surgery	Mitral valve replacement	Aortic and mitral valve replacement	Aortic valve replacement	Aortic and mitral valve replacement
Type of valve replacement	/	Medtronic Hanko II 27# (mitral valve); Medtronic Hanko II 21# (aortic valve)	/	Edward 25# (mitral valve); Edward 21# (aortic valve)
Possible predisposing factor for infective endocarditis	Smoking	Fungemia Hypertension Smoking	/	Hepatitis B
Symptoms on admission	Aggravated shortness of breath for one month and edema of both lower extremities for 3 days	Fever for 20 days and shortness of breath for 3 days after activity	Repeated fever for more than 50 days and acute bloating for 12 days	Repeated fever for nearly 2 months
Infection site	Mitral valve	Mitral valve	Aortic valve	Mitral valve
Time post implantation	Five years	Six months	Four years	Three years
Pathogen (isolated from blood)	*Candida parapsilosis *sensu stricto	*Candida parapsilosis *sensu stricto	*Candida parapsilosis *sensu stricto	*Candida metapsilosis*
MIC (ug/mL)				
5-Flucytosine	4	4	4	4
Amphotericin B	0.5	0.5	0.5	0.5
Voriconazole	0.06	0.06	0.06	0.06
Itraconazole	0.125	0.125	0.125	0.125
Fluconazole	1	1	1	2
Choice ofantifungal drugs	Voriconazole, caspofungin	Voriconazole, caspofungin, amphotericin B	Voriconazole, fluconazole	Fluconazole, caspofungin
Outcome	Successful medical therapy	Successful medical therapy	Death	Death

*MIC* minimal inhibitory concentration

### Case report 3

**Fig. 3:**
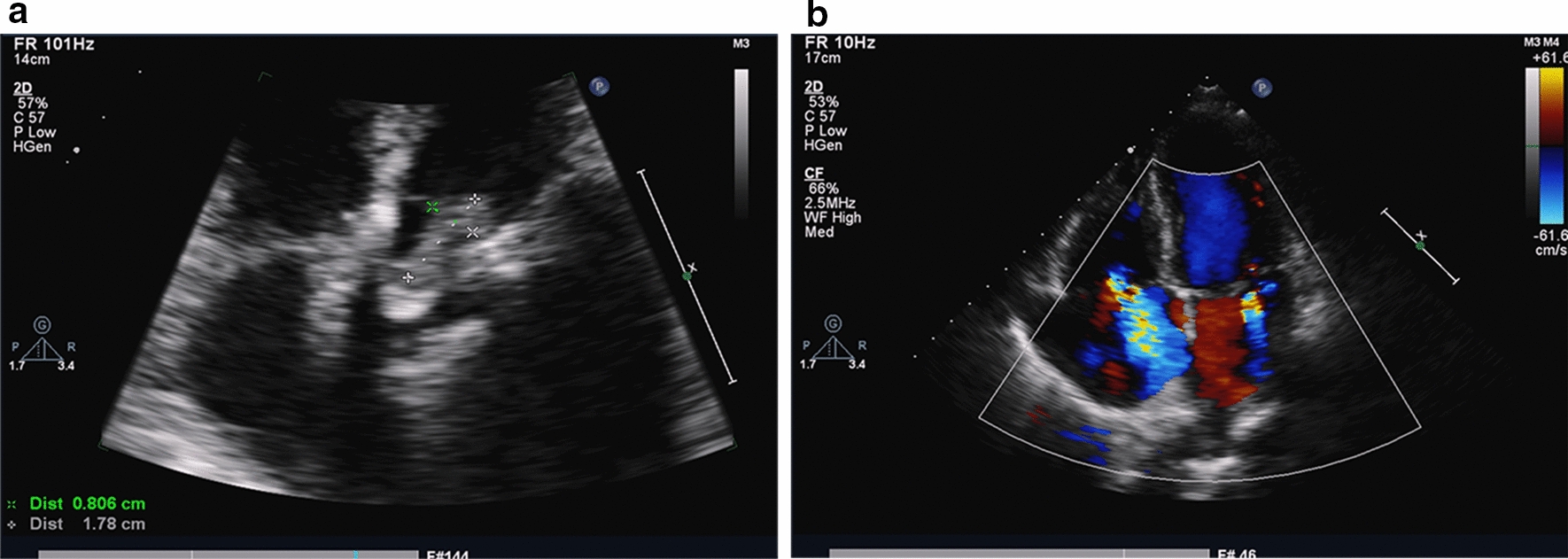
The *Candida parapsilosis* complex prosthetic valve endocarditis of case 3. **a** TTE showed a hypoechoic vegetation on the artificial aortic valve and the vegetation was about 17.8×8.0 mm; **b** Mild to moderate mitral regurgitation and moderate tricuspid regurgitation were observed on Doppler color view

The third patient was a 70-year-old man who underwent aortic valve replacement in 2011 and was admitted in the First Affiliated Hospital of Sun Yat-Sen University on November 22 of 2015 due to repeated fever for more than 50 days and acute bloating for 12 days. The initial blood count showed WBC count of 2.95 × 10^9^ cells/L (64.8% neutrophils, 22.7% lymphocytes). The PCT, troponin T, NT-proBNP and the (1,3)-β-D glucan all increased. (Table S1). The TTE showed a hypoechoic vegetation was formed on prosthetic aortic valve and the size of the vegetation was about 17.8 × 8.0 mm. Besides, enlarged left atrium and left ventricle, mild-moderate mitral valve regurgitation, moderate tricuspid regurgitation, moderate pulmonary artery hypertension was found. The LVEF was about 74% and diastolic function of left ventricular was reduced (grade I) (Fig. 3). Abdominal examination showed gas accumulation in the intestine, indicating intestinal obstruction. The patient presented septic shock, poor heart function and arrhythmia, incomplete intestinal obstruction and water and electrolyte balance disorders. The blood culture grew *Candida parapsilosis *sensu stricto (Flucytosine MIC 4 µg/mL, amphotericin B MIC 0.5 µg/mL, voriconazole MIC 0.06 µg/mL, itraconazole MIC 0.125 µg/mL, fluconazole MIC 1 µg/mL). The patient was given anti-infection and anti-shock treatment. The patient was administrated fluconazole 100 mg iv once a day from November 23 to November 27 and voriconazole 200 mg iv twice a day. Besides, the patient was also given cefperazone-sulbactam 3 g iv three times a day from November 22 to December 4. Fourteen days after a series of treatments, the patient presented cardiac rhythm of 130 beats/min, atrial fibrillation rhythm, blood pressure of 100/60 mmHg, maximum temperature 40 ℃, 30 breaths/min, SPO2 84–90% and SPO2% 90–94 under mask oxygen inhalation. The patient finally died in spite of a series of emergency measures.

### Case report 4

The fourth patient was a 64 year-old man with a history of hepatitis B and underwent aortic bioprosthesis valve replacement and mitral bioprosthesis valve replacement in 2016. Three years later, the patient was readmitted into the First Affiliated Hospital of Sun Yat-Sen University on November 7 of 2019 because of repeated fever for nearly 2 months without incentive. The initial blood count showed WBC count of 3.91 × 10^9^ cells/L (73.7% neutrophils, 18.8% lymphocytes). The PCT, troponin T and NT-proBNP were 0.61 ng/mL, 0.059 ng/mL and 10,864 pg/mL respectively, which were all increased. Bedside echocardiography showed the prosthetic mitral valve functioned normally, but there was a vegetation which was nearly 19 × 10 mm. The blood culture grew *candida metapsilosis* which was identified by MALDI TOF–MS (bioMerieux, France) (Flucytosine MIC 4 ug/mL, amphotericin B MIC 0.5 ug/mL, voriconazole MIC 0.06 ug/mL, itraconazole MIC 0.125 ug/mL, fluconazole MIC 2 ug/mL) (Table 1 and Additional file 1: Table S1). Then the patient was administrated fluconazole 100 mg iv once a day from November 7 to 9 and caspofungin 50 mg iv once a day from November 9 to 10, as well as cefperazone-sulbactam 3 g iv three times a day for four days. On the fourth day of admission, the patient had a ventricular fibrillation suddenly. After a series of rescue measures, the patient’s condition didn’t improve and died.

## Discussion

With the increasing infection rate of *Candida*, *Candida* endocarditis especially *Candida parapsilosis* complex endocarditis gains more and more attention because of its high mortality and morbidity [10, 11]. In recent years, with the widespread application of life support systems, *Candida parapsilosis* complex has become the second most common pathogen of candidiasis. *Candida parapsilosis* complex is the normal flora colonizing on gastrointestinal tract, skin and oropharynx. The general incentives for *Candida parapsilosis* complex contain the prosthetic valves (57.4%), IV drug use (IVDU; 20%), IV parenteral nutrition (6.9%), abdominal surgery (6.9%), immunosuppression (6.4%), using broad-spectrum antibiotics (5.6%) and previous valvular disease (4.8%) [12]. *Candida parapsilosis* strains are heterogeneous. Recently, *Candida parapsilosis* is recognized as a species complex. Tavanti et al. [13]suggested to divide *Candida parapsilosis* group into three species named *Candida parapsilosis *sensu stricto, *Candida orthopsilosis* and *Candida metapsilosis*. The biological phenotypic characteristics of the three species of fungi are basically the same but they belong to different genotypes. Conventional fungal identification methods cannot distinguish between them. They can only be distinguished by Matrix-assisted laser desorption ionization-time of flight mass spectrometry (MALDI TOF–MS) or molecular biology method. In the previous reports (Table 2) [14–24], the authors all did not distinguish the three species of *Candida parapsilosis* and just described the cases as *Candida parapsilosis* PVE. However, in our report, we distinguish the three species of *Candida parapsilosis.* In our study, there were three cases of *Candida parapsilosis *sensu stricto and one case of *Candida metapsilosis.* Species of *Candida parapsilosis* complex are important pathogens of nosocomial infections and the molecular identification of *Candida parapsilosis* complex at the species level is essential for optimal treatment and study of nosocomial cross-transmission [25]. Thus, it is recommended to identify *Candida parapsilosis* complex at species level.

**Table 2:** Cases and clinical management of *Candida Parapsilosis* complex prosthetic valve endocarditis

Age/Sex	Interval	Diagnosis		Initial therapy		Maintenance therapy	Survive	Author and references
		Blood culture	Culture from focus	Antifungal drugs	Surgery			
55/F	22 days	+		/	/	/	No	Otaki et al [14]
67/M	/	+	/	amphotericin B; flucytosine	Yes	Oral fluconazole 400 mg daily	Yes	Darwazah et al [15]
59/M	11 months	+	+	amphotericin B, flucytosine	Yes	Fluconazole, 200 mg daily, for six months	Yes	Jones et al [16]
82/M	5 months	/	+	7.6 g AmBisome	Yes	/	No	Jones et al [16]
54/M	/	/	+	amphotericin B fluconazole 5-flucytosine	Yes	Amphotericin B and flucytosine	Yes	Kumar et al [17]
44/F	6 weeks	+	/	/	/	/	/	Mvondo, et al [18]
36/M	10 months	/	+	caspofungin, amphotericin B	Yes	Oral antifungal suppres-sive therapy	Yes	Pepe et al [19]
35/F	3 years	/	+	Amphotericin B,	Yes	/	No	Shokohi et al [20]
47/M	2 years	+	/	amphotericin B, 5- flucytosine, micafungin	Yes	Suppressive therapy with fluconazole	Yes	Silva-Pinto et al [21]
31/F	/	+	+	Amphotericin B	Yes	Long-term prescription of fluconazole	Yes	Kabach et al [22]
69/M	/	+	/	amphotericin B, micafungin, fluconazole	No	Chronic suppression with fluconazole	Yes	Ahuja et al [23]
45/M	> 10 years	+	/	amphotericin B, micafungin, fluconazole	No	Long-term prescription of flucytosine and fluconazole	Yes	Ahuja et al [23]
24/F	5 years	+	/	/	Yes	Oral caspofungin and daily aspirin	Yes	Tan et al [24]
55/M	5 years	+	/	Voriconazole, caspofungin	No	/	Yes	Our report
71/M	6 months	+	/	Voriconazole, caspofungin, amphotericin B	No	Caspofungin 50 mg once a week and amphotericin B 30 mg once a week	Yes	Our report
70/M	4 years	+	/	Voriconazole, fluconazole	No	/	No	Our report
64/M	3 years	+	/	Fluconazole, caspofungin	No	/	No	Our report

Thanks to the development of matrix-assisted laser desorption ionization time-of-flight mass spectrometry (MALDI-TOF MS), it becomes easy, fast and convenient to identify *Candida parapsilosis* at the species level. In our report, we identified the *Candida parapsilosis* at the species level by MALDI-TOF MS quickly and accurately. Most of previous reports (Table 2) did not give out definite method to identify the *Candida parapsilosis* and they also did not identify it at the species level. Usually, conventional phenotypic identification techniques and gene sequencing are the main methods used to identify the microorganisms in clinical microbiology laboratories. With the development of MALDI-TOF MS, this technology has been adopted in clinical microbiology and it tends to be a gold standard for microbial identification [26]. On the one hand, MALDI-TOF MS can give accurate identification of most Gram-positive, Gram-negative bacterial strains and yeast isolates at the species level [27]. On the other hand, it is easy, fast, cheap, and efficient. This technology identifies each microorganism according to the analysis of mass spectra of the microorganism’ protein. By comparing the mass spectrum of the identifying microorganism with that in database of reference spectra, the microorganism could be identified at the family, genus, or species level [28]. Moreover, we usually just need pick a colony from a culture plate to spot on the target plate and add the matrix to the spot. After drying, the target plate should be taken to the mass spectrometer’s ionization chamber and the analysis can be finished within a few minutes. MALDI-TOF MS as an easy, fast and efficient identification method has advantages over conventional methods and owns the potential to influence clinical diagnostics and microbial research in the future, making it worth promoting.

No definitive treatment is recommended for *Candida parapsilosis* complex PVE and consensus on the best medical treatment and on its duration is limited. In recent years, the therapy tends to become combination antifungal therapy and the use of echinocandin is increasing [29, 30]. The echinocandins are newly developed class of synthetic antifungals which could inhibit the synthesis of 1,3-β-D-glucan synthase noncompetitively. Moreover, echinocandins can be used against the fluconazole-resistant *Candida parapsilosis* complex. Although the *Candida parapsilosis* complex may have echinocandin resistance but it is uncommon. In our case series, the combination antifungal therapy for them were mainly the combination of azoles and echinocandins and the combination antifungal therapies for them during period of hospitalization were shown in Table 3. Moreover, the therapy obtained good outcomes in the patients who came to the hospital earlier, indicating early diagnosis and prompt antibiotic therapy were essential during the treatment of *Candida parapsilosis* complex PVE. Usually, the clinical features of *Candida parapsilosis* complex PVE are non-specific. In our case series, the patients only presented fever or shortness of breath. Case 1 and case 2 came to see a doctor within one month when the symptoms appeared while case 3 and case 4 saw the doctor 2 months later. The outcomes of case 1 and case 2 were successful medical therapy while the conditions of case 3 and case 4 deteriorated, making it important to diagnose *Candida parapsilosis* complex PVE as early as possible. Besides, in the previous reports, the case in Tan’s report was admitted one month after initial symptoms and the case in Silva-Pinto’s report was admitted ten days after initial symptoms [21, 24]. These cases all had a successful medical therapy. However, the case of Shokohi who was admitted 35 days after initial symptoms died after a series of emergency measures [20]. The other cases didn’t give out definite time point. In fact, the cases concerning the *Candida parapsilosis* complex prosthetic valve endocarditis are relatively few and some cases didn’t present them in detail. Although the reasons for the outcomes of the patients varied, it is undeniable that early diagnosis influences the outcomes of the patients from the limited cases. Fungus is opportunistic pathogen and fungal infections are an important cause of morbidity and mortality in the immunocompromised population. On the one hand, the therapy that could reduce immunity such as the use of glucocorticoid may increase the risk of *Candida parapsilosis* complex PVE in patients who underwent valve replacement. On the other hand, the physical conditions of the patients could influence the risk of *Candida parapsilosis* complex PVE. When treating immunocompromised patients who had valve replacement, we should watch out for *Candida parapsilosis* complex PVE.

**Table 3:** Combination antifungal therapy during period of hospitalization

	Admission time	Hospital stays	Antifungal drugs	Period	Administration	Antifungal drugs	Period	Administration	Antifungaldrugs	Period	Administration
Case 1	2017/12/24	12 days	Voriconazole	12–25 to 12–28	200 mg IV Q12H	Caspofungin	12–28 to 1–8(2018)	50 mg Q12H iv	/	/	/
Case 2	2019/11/4	31 days	Voriconazolerr	11–4 to 11–21	200 mg IV Q12H	Caspofungin	11–21 to 12–4	50 mg Q12H iv	Amphotericin B	11–26 to 12–4	1 mg IV QD
Case 3	2015/11/22	14 days	Fluconazole	11–23 to 11–27	100 ml IV QD	Voriconazole	11–27 to 12–4	200 mg IV Q12H	/	/	/
Case 4	2019/11/7	4 days	Fluconazole	11–7 to 11–9	100 ml IV QD	Caspofungin	11–9 to 11–10	50 mg QD iv	/	/	/

Through up-to-date review of all previous cases, 12 cases of *Candida parapsilosis* complex prosthetic valve endocarditis had been reported (Table 2). Besides, two articles described outbreaks of *Candida parapsilosis* prosthetic valve endocarditis following cardiac surgery, including 8 dead cases and one alive case without detailed information [31, 32]. Among the listed cases including our cases, 9 cases both chose antifungal drugs and valve replacement to treat *Candida parapsilosis* complex prosthetic valve endocarditis while the treatment for it tended to combination antifungal therapy rather than surgery in the Ahuja’s cases. The literature about *Candida parapsilosis* complex PVE is limited. Moreover, most of previous reports did not give out detail description of their cases. Thus, more literature with detailed description about *Candida parapsilosis* complex PVE and the management of it is needed, providing reference to clinicians when treating *Candida parapsilosis* complex PVE.

In summary, our study raises significant learning points about the medical management and combination antifungal therapy for *Candida parapsilosis* complex PVE, providing reference for the treatment of *Candida parapsilosis* complex PVE to other clinicians. These cases also emphasize the challenges when treating *Candida parapsilosis* complex PVE. Besides, it is recommended to identify *Candida parapsilosis* complex at species level. MALDI-TOF MS as an easy, fast and efficient identification method is worth promoting in clinical microbiology diagnostics and microbial research in the future. Early diagnosis and prompt antibiotic therapy may play a role in the treatment for *Candida parapsilosis* complex PVE and more attention should be paid to the immunocompromised patients who underwent valve replacement.

## Supplementary Information

**Additional file 1: Table S1.** Routine analysis of blood, infectious and cardiac function indexes, and indicators of coagulation function on the day of admission.

## Data Availability

Not applicable.
