# Supplementary material for: A case series of medically managed Candida parapsilosis complex prosthetic valve endocarditis
Source: Ann Clin Microbiol Antimicrob. 2021 Jan 5;20:1. doi: 10.1186/s12941-020-00409-4 (PMC7786486; doi:10.1186/s12941-020-00409-4)
Supplement: Supplementary file 1 — Additional file 1: Table S1. Routine analysis of blood, infectious and cardiac function indexes, and indicators of coagulation function on the day of admission. [file 12941_2020_409_MOESM1_ESM.docx]

**Table S1** [**Routine**](javascript:;) [**analysis**](javascript:;) [**of**](javascript:;) [**blood**](javascript:;)**, infectious and cardiac function indexes, and indicators of coagulation function on the day of admission.**

|  | Case 1 | Case 2 | Case 3 | Case 4 |
| --- | --- | --- | --- | --- |
| WBC (cells/L) | 5.28 | 3.76 | 2.95 | 3.91 |
| NE% | 46.6 | 78.8 | 64.8 | 73.7 |
| LY% | 36.2 | 10.3 | 22.7 | 18.8 |
| PLT (counts/L) | 237 | 73 | 118 | 48 |
| RBC (cells/L) | 4.55 | 2.67 | 3.32 | 3.64 |
| HGB (g/L) | 100 | 89 | 92 | 104 |
| PCT (ng / mL) | 0.39 | 0.66 | 0.5-2.0 | 0.61 |
| (1,3) -β-D glucan (pg / ml) | 93.9 | 144.85 | 824 | / |
| TNT (ng / mL) | 0.015 | 0.062 | 0.011 | 0.059 |
| Pro-BNP (pg / mL) | 2328 | 4632 | 4637 | 10864 |
| APTT(s) | 20.8 | 47.3 | 42.9 | 36.2 |
| INR | 3.3 | 1.71 | 1.47 | 1.32 |
| PT(s) | 35.9 | 19.1 | 17.4 | 15 |

WBC: white blood cell; NE: neutrophil; LY: lymphocyte; PLT: platelet; RBC: red blood cell; HGB: hemoglobin; PCT: [procalcitonin](javascript:;); TNT: troponin T; Pro-BNP: N-terminal prohormone of brain natriuretic peptide; APTT, Activated partial thromboplastin time; PT, Prothrombin time.
